# Application of artificial intelligence in geriatric infection: recent advances and prospects

**DOI:** 10.3389/fcimb.2026.1887322

**Published:** 2026-08-13

**Authors:** Puyu Liang, Zhaoyi Tan, Beibei Liang, Yuxi Xu, Tianlin Wang, Zhijian Zhang, Yun Cai

**Affiliations:** 1 The Phase I Clinical Trial Laboratory, the First Medical Center of the PLA General Hospital, Beijing, China; 2 Center of Medicine Clinical Research, Department of Pharmacy, Medical Supplies Center, PLA General Hospital, Beijing, China; 3 Department of Pulmonary and Critical Care Medicine, The Second Medical Center and National Clinical Research Center for Geriatric Diseases, Chinese PLA General Hospital, Beijing, China

**Keywords:** artificial intelligence, clinical therapies, diagnosis, early warning, geriatric infection

## Abstract

**Background:**

As global population aging accelerates, infectious diseases in older adults have emerged as a growing public health burden with substantial clinical, economic, and societal implications. The rapid advancement of artificial intelligence (AI) offers a transformative opportunity to enhance the management of infections in this vulnerable population.

**Methods:**

This review delineates key determinants specific to geriatrics that influence infection susceptibility and atypical presentation, including age-related organ dysfunction, immunosenescence, inflammaging, chronic comorbidities, and psychosocial factors, and summarizes the recent advances in AI applications across the management for infectious diseases in older adults.

**Results:**

AI has made significant progress in the prevention, diagnosis, and treatment of infectious diseases in older adults: early detection of clinical deterioration, rapid and etiologically precise diagnosis, individualized therapeutic optimization, AI-facilitated antimicrobial stewardship, and accelerated discovery of novel antimicrobial strategies.

**Conclusion:**

AI shows great potential in helping prevent, diagnose, and treat infections in older adults, and represents a promising complement to traditional care approaches.

## 1 Introduction

Global population aging is an unprecedented demographic trend (Noren Hooten et al., 2022; de Magalhães, 2025). By 2050, the number of individuals aged 65 years and older is projected to reach approximately 1.6 billion, accounting for nearly 16.6 percent of the world’s population (Lutz et al., 2008; He et al., 2016). Due to demographic shifts, the morbidity and mortality of infections among the elderly have increased (Ramírez-Soto, 2023). Moreover, immunosenescence, chronic comorbidities, and a decline in physiological reserves all put elders at greater risk of contracting and ineffectively fighting infections (Vetrano et al., 2021). Consequently, the management of elderly patients with infections is a distinct and increasingly critical domain of geriatric medicine (Ibarz et al., 2024). Antimicrobial therapy has long been considered as a cornerstone in the treatment of infections, while elderly patients often require individualized anti-infective treatment regimens to ensure safety and efficacy (Butranova et al., 2023; Fu et al., 2024). Although multiple current guidelines recommend therapeutic drug monitoring (TDM) of antibiotics in elderly patients, existing management models still face several challenges (Abdul-Aziz et al., 2020). For example, infections in the elderly often present with atypical symptoms, such as the lack of fever or altered mental status, which can obscure the clinical picture and lead to delayed diagnosis or inadequate treatment (Christ and Heppner, 2009; Waalen and Buxbaum, 2011). In addition, the distribution of pathogenic microorganisms in elderly patients differs significantly from that in younger populations, leading to differences in optimal empiric antimicrobial coverage for serious infections (Alves et al., 2024; Mancinetti et al., 2024). These points highlight the necessity of introducing innovative technologies.

In recent years, artificial intelligence (AI)-driven approaches, including machine learning, deep learning, and image recognition, have been increasingly applied across the full spectrum of infectious disease care. Srivastava et al. provided a comprehensive overview of AI in the early diagnosis and treatment of infectious diseases, highlighting how AI-powered diagnostic tools enhance disease detection accuracy, how predictive models enable proactive outbreak management, and how AI accelerates the drug discovery process (Srivastava et al., 2025). Building on these advances, AI holds particular promise for optimizing the individualized diagnosis and treatment of infections in older adults, a population whose clinical complexities demand precisely the multidimensional data integration and predictive capabilities that AI offers. AI integrates multidimensional clinical data to enable early and accurate diagnosis (Garcés-Jiménez et al., 2024), leverages algorithmic models to predict drug efficacy and adverse reactions; and dynamically adjusts medication regimens, thereby directly addressing key limitations of traditional management approaches and creating new avenues for improving prognosis in elderly patients with infections (Pinto et al., 2025).

This review systematically synthesizes evidence on applications of AI in the management of infections among older adults, identifies approaches driven by AI that have promising translational potential for geriatric infection care, and aims to inform novel, evidence informed strategies for improving clinical outcomes in this population.

## 2 Methods

We primarily searched for literature published between January 2020 and June 2026 in PubMed and Web of Science. Search terms: (“Infectious Disease” OR “Infection” OR “Sepsis” OR “Pneumonia” OR “Antimicrobial Resistance” OR “Drug Resistance”) AND (“Artificial Intelligence” OR “Machine Learning” OR “Deep Learning” OR “Neural Network” OR “Large Language Model”). To ensure the methodological rigor and relevance of this narrative review, specific exclusion criteria were applied. Studies were excluded if they: (1) did not involve AI or machine learning–based methodologies; (2) did not address infectious diseases or infection-related clinical outcomes; (3) were editorials, letters, commentaries, or conference abstracts without full-text availability; (4) were published in languages other than English; or (5) were duplicates of previously identified records.

## 3 Physiopathological factors associated with infectious diseases in the elderly

In the elderly, age related organ specific physiologic changes, immunosenescence and inflammaging, comorbidities, and their personal attitudes toward quality of life all influence the clinical presentation of infections. Understanding the influence of all these changes may facilitate the prevention, diagnosis and treatment of infections in the elderly.

### 3.1 Age-related organ dysfunction

During the process of physiological aging, multiple organ systems critical to the response against infection are affected (**Table 1**), independent of any existing comorbidities or diseases. These changes alter the patient’s immune and defense status as well as physiologic stress response, potentially contribute to vulnerability to infections. In the respiratory system, aging leads to weakened cough reflexes, reduced mucociliary clearance, altered respiratory mechanics, and decreased respiratory tract immunoglobulin levels, collectively resulting in a significant increase in pneumonia incidence (Janssens and Krause, 2004; Angulo et al., 2016; Bailey, 2022; Hill et al., 2022; Kageyama et al., 2024; Kroemer et al., 2025). For the digestive system, aging-induced alterations encompass multifaceted functional and microbiological changes from the oral cavity to the intestines, markedly elevating susceptibility to gastrointestinal infections in the elderly (Katelaris et al., 1993; Dodds et al., 2005; Hara et al., 2019; Chen et al., 2020; Saint-Criq et al., 2021; Zheng et al., 2022; Qiu et al., 2025). Regarding the urinary system, aging primarily manifests as impaired bladder function, urodynamic abnormalities, and pelvic floor muscle relaxation, which collectively heighten the risk of urinary retention and consequently substantially raise the prevalence of urinary tract infections in the older population (Rodriguez-Mañas, 2020; Brunker et al., 2023). In the context of the skin, aging is accompanied by structural weakening of the dermis and diminished blood supply, compounded by reduced mobility, together increasing the risk of skin and soft tissue infections as well as secondary infections such as pressure injuries in the elderly (Tieland et al., 2018; Falcone and Tiseo, 2023).

**Table 1:** Age related organ-specific physiologic changes.

Affected organ system	Age-related physiological changes	Increased susceptible infections
Respiratory System (Janssens and Krause, 2004; Angulo et al., 2016; Kroemer et al., 2025)	1.Age-related blunting of cough and other airway-protective reflexes 2. Impaired mucociliary clearance, leading to retention of pathogens and foreign bodies in the airways 3.Decline in respiratory muscle strength, chest wall compliance, and static elastic recoil 4.Decreased concentration of immunoglobulins in respiratory secretions	Pneumonia (incidence 4–5 times higher than that in young and middle-aged adults) (Bailey, 2022; Hill et al., 2022; Kageyama et al., 2024)
Gastrointestinal and Digestive Systems (Katelaris et al., 1993; Dodds et al., 2005; Hara et al., 2019; Chen et al., 2020; Saint-Criq et al., 2021; Zheng et al., 2022; Qiu et al., 2025)	1.Intestinal functional aging accompanied by increased mucosal barrier permeability, gut dysbiosis, and impaired metabolic capacity 2.Reduced salivary secretion and alterations in antimicrobial proteins within saliva 3.Weakened tongue muscle strength and slowed swallowing speed 4.Decreased gastric acid secretion (attributed to gastric mucosal atrophy, proton pump inhibitor use, or surgery) 5. Impaired intestinal motility 6. Imbalances in resident intestinal flora 7.Delayed recovery of gut microbiota following antimicrobial administration	Bacterial enterocolitis (Saint-Criq et al., 2021)
Urinary System (Rodriguez-Mañas, 2020)	1.Mechanical alterations: reduced bladder capacity, decreased urinary flow rate, increased post-void residual volume 2. Enhanced bacterial adhesion of urothelial cells 3. Bladder prolapse (females) and prostatic diseases (males) contributing to urinary stasis 4. Decline in estrogen levels (postmenopausal women) 5. Decreased muscle fiber count, elastic fiber degeneration, reduced muscle contractility and endurance	UTIs (prevalence up to 15%-20% in elderly women) (Brunker et al., 2023)
Skin and Musculoskeletal System (Falcone and Tiseo, 2023)	1. Collagen loss and reduced dermal blood vessels leading to thinner, weaker skin 2. Pathologic conditions (edema or trauma) in the elderly 3. Declining musculoskeletal function reducing mobility, resulting in prolonged bed rest	1. Skin and soft tissue infections (cellulitis, erysipelas, necrotizing fasciitis) 2. Pressure ulcers (Tieland et al., 2018)

UTIs, Urinary Tract Infections.

### 3.2 Age-related immunosenescence and inflammaging

The aging process is accompanied by qualitative and quantitative changes in the immune system (El Chakhtoura et al., 2017; Cunha et al., 2020; Li et al., 2025). Specifically, it refers to the comprehensive structural remodeling and functional decline of the immune system associated with advancing age, representing an adaptive adjustment of the immune system to the aging microenvironment rather than a mere functional impairment (Huang et al., 2021). This process, also called immunosenescence, is characterized by the synergistic impairment of both innate immunity and adaptive immunity (Paul, 2011; Ajoolabady et al., 2024). Immunosenescence significantly weakens the ability of the elderly to resist pathogens, resulting in an infection risk that is 2–3 times higher than that of young and middle-aged adults (Santoro et al., 2021). This constitutes a key mechanism underlying the heightened susceptibility to infection in the elderly (Quiros-Roldan et al., 2024).

At the level of innate immunity, natural killer (NK) cells exhibit impaired perforin release and defective granule exocytosis (Mujal et al., 2021). The chemotaxis, bactericidal capacity, phagocytic activity, reactive oxygen species (ROS) production, and neutrophil extracellular trap (NET) formation ability of neutrophils are compromised (Kanashiro et al., 2020). The antigen presentation ability and cytokine secretion balance of macrophages are dysregulated (Brorson et al., 2025). At the level of adaptive immunity, thymic involution results in reduced production of naïve T cells (Zhang et al., 2021). The ratio of naïve T cells to memory T cells in peripheral blood is reversed, and the CD4^+^/CD8^+^ T cell ratio is disrupted (Liu et al., 2023). The clonal diversity of memory T cells is drastically reduced, with a subset exhibiting a senescent phenotype (Sun et al., 2022). The secretory function of key cytokines such as IL-2 (Fu et al., 2025) and IFN-γ is impaired. In terms of B cells (Marth et al., 2002), aging is associated with reduced antibody repertoire diversity, impaired class-switch recombination, and diminished antibody production efficiency (Hou et al., 2024). In addition, the abnormal expression of co-inhibitory receptors (e.g. PD-1, Tim-3, TIGIT) on the surface of immune cells exacerbates T cell exhaustion, which further impairs the efficacy of immune responses (Fukushima et al., 2025).

Immunosenescence is accompanied by complex immune dysregulation (Ajoolabady et al., 2024). Specifically, reduced dNTP pool availability promotes erroneous rNTP incorporation during mitochondrial DNA (mtDNA) synthesis, rendering mtDNA structurally unstable and prone to double-strand breaks and subsequent cytosolic release. This persistent mtDNA leakage chronically activates the cGAS-STING pathway, driving sustained inflammation that further impairs immune cell function, thereby establishing a self-amplifying cycle of infection driven inflammation (Bahat et al., 2025). Inflammaging, is a state of chronic low-grade inflammation associated with aging, triggered by sustained endogenous or exogenous antigen stimulation (Li et al., 2023; Liu et al., 2023). Its core manifestation is a sustained elevation in circulating levels of pro-inflammatory mediators, such as IL-6, TNF-α, and C-reactive protein (CRP), alongside impaired anti-inflammatory regulation. This chronic immune dysregulation disrupts inflammatory homeostasis, thereby compromising the efficient clearance of pathogens and senescent cells (Khan et al., 2024). Additionally, sustained inflammatory signals damage tissues and organs, promoting the development of age-related conditions including frailty syndrome and atherosclerosis (Rasa et al., 2022). Inflammaging and immunosenescence form a vicious cycle. Specifically, immunosenescence facilitates the onset of inflammaging by impairing the body’s ability to clear pathogens. Meanwhile, inflammaging in turn inhibits immune cell function via persistent inflammatory signaling, which further accelerates the progression of immunosenescence (Nguyen and Cho, 2025). Notably, both immunosenescence and inflammaging in elderly patients are regulated by factors such as nutritional status, obesity, and frailty (Bosco and Noti, 2021; Chen et al., 2022).

### 3.3 Chronic comorbidities

The geriatric patient is characterized by chronic comorbidities/multimorbidity. The most significant comorbidities triggering infections are diabetes and chronic heart failure. Data shows that the incidence of sepsis after infection in elderly patients with comorbid diabetes is 1.8 times that of non-diabetic patients. Moreover, when blood glucose control is poor (with glycated hemoglobin > 8.5%), the infection risk further increases (Hepper et al., 2013; Alexander et al., 2024; GBD 2021 Nervous System Disorders Collaborators, 2024). It is noteworthy that there are differences in the expression level of ACE2 in heart tissue between the elderly and young populations, which is indirectly associated with the efficacy of immune response in patients with heart disease. Ultimately, this increases the risk of severe infection in elderly patients with comorbid heart disease and COVID-19 (Koff and Williams, 2020).Besides the two diseases mentioned above, Chronic Kidney Disease (CKD) has also been confirmed to impair immune function and lead to infections. Specifically, CKD leads to dysfunction of antigen-presenting cells (CD80/CD86 deficiency), reduced expression of Toll-like receptor 4 (TLR4), and other effects. It also causes erythropoietin (EPO) deficiency and accumulation of uremic toxins, which damage immune cells and thereby disrupt adaptive immune regulation (Hou et al., 2021).

### 3.4 Psychosocial factors

Psychological issues in the elderly, such as loneliness and depression, can regulate immune function and inflammatory levels through the neuroendocrine immune axis (Hoang et al., 2022). Loneliness continuously activates the hypothalamic-pituitary-adrenal (HPA) axis, leading to chronic elevation of cortisol levels. Excessively high cortisol can inhibit T cell proliferation and macrophage phagocytic function, impairing the immune clearance capacity (Finley and Schaefer, 2022). Studies have shown that the incidence of respiratory infections in elderly people living alone for a long time is 37% higher than that in socially active counterparts, and the duration of symptoms after infection is prolonged by 2–3 days (Bu et al., 2020). In addition, anxiety and stress responses in the elderly can also affect infection outcomes through short-term immune dysregulation (Slavich and Irwin, 2014; Alotiby, 2024).

## 4 AI in the management of infectious diseases in geriatric populations

In recent years, the rapid advancement of AI has emerged as a significant highlight of the era. Leveraging its powerful data processing, pattern recognition, and decision support capabilities (Kraemer et al., 2025), AI offers new approaches and methodologies for the prevention, diagnosis, and treatment of infectious diseases in the elderly (**Table 2**).

**Table 2:** Representative AI studies on infectious diseases in the elderly.

AI application	Author	Disease studied	AI method used	Outcome	Benefit over traditional approaches
AI in Early Warning and Prevention of Infectious Diseases	Lin(2025) Xie (2025) van der Meijden (2025)	Multiple infectious diseases Long-term COVID-19 monitoring Postoperative infection	Digital Twin Framework + AI + RT-PCR LLM based on BLOOM XGBoost + multicenter EHR data	Reconstruct unrecorded infection cycles Optimize secondary transmission control by 60% Detected infection trends 7.63 days earlier than official reports; monitored recovery cycles, reinfection rates and long-term health effects Predicted infection risks at 7 and 30 days post-surgery	Enable timely tracking of transmission chains Improve completeness of elderly infection cycle reconstruction Provided targeted support for infection prevention and control in elderly populations; enabled earlier detection than traditional surveillance Enabled early intervention to reduce disease recurrence in high-risk elderly demographic; provided reliable foundation for clinical decision-making
	Chen (2020)	SSI	AMRAMS	Predicted SSI risk in hospitalized surgical patients (nearly 30% of dataset were elderly ≥65 years)	Demonstrated significantly higher accuracy than conventional machine learning models and the NNIS risk index; optimized risk stratification for elderly surgical patients
	Gutierrez-Naranjo (2024)	SSI after lower extremity fracture surgery	ML	Predicted SSI risk with AUC of 77.8%; identified 5 key predictors: operating room duration, ankle region involvement, open injury	Enabled surgeons to identify high-risk elderly patients with lower extremity fractures; facilitated targeted prevention of complications
	Jakobsen (2024)	HA-UTI	ML(neural network + SHAP method)	Identified HA-UTI risk within 24h of admission; optimal model achieved AUC 0.758; enabled population- and individual-level interpretability	Rapidly detected HA-UTI risk in elderly patients; interpretability helped clinicians understand risk sources, enabling targeted early prevention and reducing hospital-acquired infection incidence in the elderly
	Shashikumar (2025)	Sepsis	AI + Large Language Model	Increased positive predictive value from 22.6% to 52.9%; reduced	Captured nuanced clinical context from unstructured EHR texts, which aligns with atypical infection
	Lauritse (2020)	Sepsis	DL-based CNN-LSTM model	Provided early warning 3 hours prior to sepsis onset; learned feature interactions from raw event sequence data	Eliminated reliance on preprocessed features; improved accuracy in identifying complex infection scenarios in the elderly; supported rational medical resource allocation
	Ghosh (2013)	Hospital-acquire d infections	Ward-based intelligent hand hygiene training system	Significantly improved hand hygiene compliance and technique in clinical settings	Strengthened hospital infection control by optimizing medical staff training; played a key role in early warning and management of infections in the elderly
AI in Disease Early Warning	Zaslavsky (2025)	Infectious diseases and autoimmune diseases	DL	Accurately diagnose 6 diseases comprehensively	Reduces the invasiveness and time cost of detection
	Christians (2024)	Drug-resistant infectious diseases	Probit model + AMR statistical model + homologous sequence exclusivity assessment + plasma mcfDNA sequencing	Achieve rapid AMR result output within 1–3 days Improve PPA from 55.5% to 83.3%	Avoid invasive procedures Reduce complication risks for immunocompromised elderly
	Maturana (2022)	Malaria	AI-based digital imaging tools	Rapidly classified, detected, and quantified Plasmodium parasites in blood smears	Provided a more efficient alternative to traditional optical microscopy; highly suitable for wide clinical application, which is critical for elderly patients requiring accessible diagnostic tools
	Wang (2023)	Pneumonia	DL model	Rapidly and accurately classified pneumonia types from CT images; achieved sensitivities of 0.694 and 0.580	Diagnostic performance significantly superior to radiologists; supports timely and precise identification of pneumonia in elderly patients, who are at high risk for severe respiratory infections
	Shao (2024)	Pneumonia	MMI system Transfer learning + retrained transformer	Accurately differentiate pneumonia from other diseases; Precisely predict pulmonary infectious diseases & their etiological types	Higher accuracy in disease differentiation; Precise etiological prediction
	Jacot (2024)	Bacterial infections	AI-based image processing	Detected bacterial growth as early as 4–6 hours; at 48h, achieved 99.80% sensitivity and 91.97% specificity; at 24h, 99.08% sensitivity and 93.37% specificity	Provided early bacterial growth detection, gaining valuable time for clinical intervention; reduced workload of microbiologists by automating sterile plate processing, which is critical for timely care in elderly patients
	Burns (2023)	Bacterial infections	IAAI	Detected bacterial colonies, parasites, and mycobacteria; supported score-based classification	Streamlined diagnostic workflows in clinical microbiology; enhanced efficiency and quality of pathogen identification, which is critical for timely care in elderly patients with infections
	Hasan Pour (2025)	Superficial fungal infections	AI-based image analysis	Summarized 54 studies; AI supported diagnosis, treatment, and prevention of superficial mycoses; enabled classification of fungal pathogens	Complemented clinician skills and laboratory results; addressed limitations of time-consuming traditional diagnostic methods, which is critical for timely care in elderly patients with immune decline
	Xu (2023)	Clinical fungal infections	AI classification model + SCRS	Distinguished fungi from bacteria with 100% sensitivity/specificity; achieved 100% accuracy in fungal species identification; reduced diagnostic time to <1 hour for urinary tract infection samples	Eliminated reliance on time-consuming fungal culture (days to months); provided rapid, accurate diagnosis, which is critical for timely intervention in elderly patients with high-risk infections
	Turon (2025)	Malaria and tuberculosis	AI+PBPK Model	Precise drug administration in over 91% of the population	Avoid adverse reactions caused by standard-dose medication
AI in the Treatment of Infectious Diseases	Shapiro Ben David (2025)	Urinary tract infections	Three integrated modules based on AI machine learningo+HER	Formulate personalized antibiotic treatment plans	Reduce the use of broad-spectrum antibiotics
	Cavallaro (2023)	Bacterial infections	Gradient boosted decision tree + Shapley explanation model	Predicted antimicrobial drug resistance; substantially reduced mismatched treatment risk; provided interpretable associations between data and outcomes	Supports rational antibiotic use in elderly patients (who are at high risk of antibiotic resistance and adverse effects); improves trust in AI-driven decisions via explainable predictions, facilitating safer infection management
	Pruthi (2024)	Tuberculosis	Tree-based ensemble methods	Predicted first-line (AUC 88.3-96.5) & second-line (AUC 84.1-95.4) drug resistance; improved sensitivity for low-frequency variants (e.g., pyrazinamide +25%); identified novel resistance markers	Assists rapid, accurate drug resistance assessment for elderly TB patients (who are at higher risk of severe disease), supporting timely, targeted treatment and reducing ineffective therapy
	Wan (2025)	Infectious diseases	AI algorithms	Identified potential new therapeutic uses for existing drugs by analyzing large-scale datasets; revealed hidden drug-response patterns	Bypassed early drug development stages, reduced time/cost of bringing therapies to market; supports rapid access to safe, repurposed drugs for elderly patients
	de la Fuente-Nunez C (2025)	Drug-resistant infectious diseases	ML algorithms	Accelerated rapid identification of potential antibiotic candidates	Reduced time/cost of traditional antibiotic discovery; addresses antimicrobial resistance threats, providing timely new therapies for elderly patients
	Catacutan (2025)	Enterobacteriace ae infections	DL + chemical screening + CRISPRi	Discovered enterololin (BAY-524)	Avoids gut dysbiosis
	Popa (2022)	Antibiotic-resista nt bacterial infections	DL+ QSAR/ML + Raman/MALDI-TOF + NN	Shortened antibiotic preclinical phase; accelerated data curation; enabled faster identification of new antibiotics	Reduced high costs/time of traditional antibiotic development; supported rapid response to AR
	Keshavarzi Arshadi (2020)	COVID-19	ML/DL + CoronaDB-AI dataset	Identified SARS-CoV-2 molecular targets; provided dataset for training models; accelerated in silico drug/vaccine candidate discovery	Reduced cost/time of traditional drug/vaccine development; supported rapid response to pandemic
	Talat (2023)	Drug-resistant infections	AI platforms	Identified β-lactamase inhibitors & antibiotic alternatives; supported discovery of low-resistance antimicrobial candidates	Faster,cost-efficient drug development
	Ma (2022)	Drug-resistant bacterial infections	DL	Identified 2,349 gut microbiome AMP candidates; 181 synthesized AMPs showed >83% antimicrobial activity; 11 potent AMPs reduced mouse lung infection bacterial load >10-fold	Overcame short-AMP prediction challenges; accelerated novel AMP discovery (vs. traditional screening); effective against resistant pathogens (critical for high-risk elderly)
	Wang (2025)	Multidrug-resista nt bacterial infections	Protein LLM + subLLMs	Screened hundreds of millions of peptides; Mined/generated AMPs showed low resistance risk (in vitro CRAB/MRSA);	High-throughput, efficient AMP exploration
	Jiang (2025)	Drug-resistant bacterial infections	AMP-hydrogel-Desig ner	Generated thiol-containing AMP + hydrogel to form AI-AMP-hydrogel;In vitro: >99.99% bactericidal efficacy against MRSA/E. coli	Solved traditional biomaterial development’s lack of systematics/predictability;Precisely targets drug-resistant pathogens

AI, Artificial Intelligence; AMPs, Antimicrobial Peptides; AMR, Antimicrobial Resistance; AMRAMS, Artificial Intelligence-based Multimodal Risk Assessment Model for Surgical Site Infection; ARGs, Antibiotic Resistance Genes; ASIR, Age-standardized Incidence Rate; AUC, Area Under the Curve; BMI, Body Mass Index; CKD, Chronic Kidney Disease; CNN-LSTM, Convolutional Neural Network-Long Short-Term Memory; CRBSI, Catheter-related Bloodstream Infections; CVC-BSI, Central Venous Catheter-related Bloodstream Infections; CT, Computed Tomography; EHR, Electronic Health Record; EPO, Erythropoietin; HAIs, Healthcare-associated Infections; HA-UTI, Hospital-acquired Urinary Tract Infection; HPA, Hypothalamic-pituitary-adrenal; KOH, Potassium Hydroxide; LLM, Large Language Model; LSTM, Long Short-Term Memory; MDR, Multidrug Resistance; MDR-TB, Multidrug-resistant Tuberculosis; ML, Machine Learning; MMI, Multimodal Medical Information; mtDNA, Mitochondrial DNA; NET, Neutrophil Extracellular Trap; NK, Natural Killer; PAS, Periodic Acid Schiff; RAG, Retrieval Augmented Generation; ROS, Reactive Oxygen Species; SHAP, SHapley Additive exPlanation; SSI, Surgical Site Infection; SVM, Support Vector Machine; TB, Tuberculosis; TDM, Therapeutic Drug Monitoring; TLR4, Toll-like Receptor 4; XDR-TB, Extensively Drug-resistant Tuberculosis

### 4.1 AI in early warning and prevention of infectious diseases

Instead of infectious signs typical of younger patients, the elderly often exhibit atypical presentations (Waalen and Buxbaum, 2011).Therefore, the issue of lag in early warning and control of traditional surveillance methods (including laboratory testing and manual tracing) becomes even more prominent, especially when confronting emerging infectious diseases(EIDs) (Meckawy et al., 2022). Lin et al. integrated AI with serial RT-PCR testing within a metaverse-enabled framework, establishing a four-tier digital twin architecture comprising physical, digital, virtual, and analytical twins. This architecture enables Ct value-guided precision in COVID-19 contact tracing and quarantine decision-making, thereby enhancing both the timeliness and accuracy of containment for emerging infectious diseases. By day 24, intervention effectiveness reached 90%. Critically, the analytical twin reconstructs unrecorded infection windows, improving secondary transmission control following contact tracing by 60% (Lin et al., 2025). Furthermore, Xie et al. developed a large language model (LLM) based on the open-source BLOOM architecture for long-term COVID-19 monitoring. The model detected infection trends 7.63 days earlier than official reports (Xie et al., 2025). It monitors recovery cycles, reinfection rates, and long-term health effects, providing targeted support for their infection prevention and control in elderly populations. Beyond general outbreaks, AI facilitates postoperative infection prediction. Van der Meijden et al (van der Meijden et al., 2025) utilized an XGBoost model combined with multicenter electronic health record (EHR) data to predict infection risks in elderly patients at 7 and 30 days post-operative. This provides a reliable foundation for early intervention, ultimately reducing the risk of disease recurrence in this high-risk demographic.

Elderly patients are a high-risk group for healthcare-associated infections (HAIs),an epidemiological study reported that prevalence reaches 11.5% among patients over 85 years, compared with 7.4% in those younger than 65 years (Cairns et al., 2011). Surgical site infection (SSI) is one of the most common HAIs in the elderly population. Chen et al. developed an AI-based Multimodal Risk Assessment Model for Surgical Site Infection (AMRAMS). The model was trained on routinely collected clinical data, with nearly 30% of the dataset consisting of patients aged 65 years or older, to predict SSI risk in hospitalized surgical patients (Chen et al., 2020). In addition, Gutierrez-Naranjo et al. developed a predictive model primarily for risk stratification of SSIs following lower extremity fracture surgery. The model was trained on clinical data from 1,579 patients (30% elderly) who underwent lower extremity fracture surgery. The analysis identified five key predictors, namely operating room duration, ankle region involvement, open injury, body mass index, and patient age. Four machine learning (ML) algorithms, including neural networks, were employed for model development. The final model demonstrated robust performance in predicting post-operative SSI risk (e.g., area under the receiver operating characteristic [ROC] curve = 77.8%), providing surgeons with a valuable tool to identify high-risk patients and facilitate the prevention of complications (Gutierrez-Naranjo et al., 2024). This model, by identifying their infection risk factors (age-linked physiological decline and surgical variables), enables surgeons to target high-risk elderly patients for proactive interventions, reducing their postoperative infection incidence and preventing complications.

In the ICU setting, the support vector machine (SVM) model has been proven to be more suitable than traditional statistical methods for the early prediction of HAI risk in elderly patients admitted to the ICU (Barchitta et al., 2021). Furthermore, elderly patients are at a high risk of catheter-related bloodstream infections (CRBSI), and the fully automated monitoring algorithm for central venous catheter-related bloodstream infections (CVC-BSI) developed based on AI has demonstrated favorable performance and can effectively replace manual monitoring (Karmefors Idvall et al., 2024). All these AI applications and ML assist healthcare providers in taking timely preventive measures and help reduce the risk of severe illness in elderly patients after infection. Jakobsen et al. developed a ML model primarily designed to identify patients at risk of hospital-acquired urinary tract infection (HA-UTI) within 24 hours of hospital admission. Trained on data from 138,560 hospital admissions in the North Denmark Region, which includes a subset of elderly patients, the optimal neural network model achieved an area under the curve (AUC) of 0.758. Notably, the SHapley Additive exPlanation (SHAP) method enabled both population- and individual-level clinical interpretability of the model. Given that older adults are a high-risk group for HA-UTI (due to factors like age-related immune decline and high rates of urinary catheterization), this model can rapidly detect HA-UTI risk in elderly inpatients (Jakobsen et al., 2024).

Sepsis is a severe systemic inflammatory response syndrome. Study indicated that the incidence rate of sepsis in adults over 65 is growing 20.4% faster than in younger and middle-aged adults under 65 (Okeke et al., 2024). Elderly patients, with their impaired immune function and high susceptibility to other infectious diseases such as pneumonia and urinary tract infections, are a high-risk group for sepsis. Shashikumar et al. leveraged AI and LLMs to develop the COMPOSER-LLM system. By fusing structured data and unstructured text from EHRs and dynamically retrieving similar cases with retrieval augmented generation (RAG) technology (Shashikumar et al., 2025), the system increased the positive predictive value from 22.6% to 52.9% and reduced the false alarm rate of early warning by 76%,with a median age of the study cohort approaching 65 years, nearly half of the participants were elderly. By identifying age-associated physiological decline and surgery-specific variables as key infection risk factors, this model enables surgeons to prioritize high-risk older adults for proactive perioperative interventions, thereby reducing postoperative infection incidence and mitigating downstream complications. In addition, Lauritsen et al. proposed a deep learning-based Convolutional Neural Network-Long Short-Term Memory (CNN-LSTM) model that provides an early warning 3 hours prior to sepsis onset. By learning feature interactions directly from raw EHR event sequence data, this model eliminates reliance on preprocessed features, addressing a key limitation of traditional models that struggle with incomplete or inconsistent clinical data. Its ability to accurately identify complex infection scenarios makes it particularly promising for older populations, as it can better capture the non-typical, subtle clinical presentations common in elderly patients, thereby supporting more rational medical resource allocation and optimizing age-tailored infection management processes (Lauritsen et al., 2020).

Beyond clinical monitoring, AI significantly bolsters hospital infection control by optimizing the professional development of medical staff. Ghosh et al. demonstrated that a ward-based intelligent hand hygiene training system significantly improves compliance and technique in clinical settings (Lotfinejad et al., 2021), these systems play a significant role in the hospital’s management and control of infection early warning for the elderly.

### 4.2 AI in infectious disease diagnosis

Conventional antimicrobial resistance (AMR) detection usually requires obtaining samples through invasive methods such as puncture, while elderly patients often exhibit poor tolerance to invasive procedures (McKelvie et al., 2019) (Mancinetti et al., 2024; Nie and Zeng, 2024). Zaslavsky et al. developed Mal-ID, a multi-class deep learning model centered on supervised learning, which can rapidly and massively obtain BCR/TCR sequences via high-throughput sequencing, more comprehensively characterize immune status (Zaslavsky et al., 2025). Besides, Christians et al. developed and established an AI-driven AMR statistical model, which integrates the probit model, AMR statistical model, and assessment of exclusivity to homologous sequences. Importantly, this statistical model incorporates AMR detection into the Karius test workflow, eliminating the reliance on bacterial culture and enabling the rapid output of drug resistance results within 1–3 days (Christians et al., 2024), streamlining workflows and reducing physical burden on the elderly.

As AI computer vision techniques have grown, so have their applications to infectious disease imaging. Malaria is particularly dangerous for the elderly, often progressing to severe forms and increasing susceptibility to co-infections (Yoboue et al., 2022). New AI-based digital imaging tools provide a powerful alternative to traditional optical microscopy. These systems can rapidly classify, detect, and quantify *Plasmodium* parasites in blood smears (Wang et al., 2023). This technology is highly suitable for wide clinical application, which is critical for elderly patients who require efficient and accessible diagnostic tools (Maturana et al., 2022). In addition, AI-based image processing has been successfully applied to the detection of MRSA, exhibiting high sensitivity and specificity. This AI model can achieve the early identification of bacterial growth within 4 to 6 hours, thereby gaining valuable time for early clinical intervention (Jacot et al., 2024).Now integrated within automated laboratory systems, AI-driven image analysis tools are being utilized to detect bacterial colonies, identify pathogens cultured on chromogenic agar, and streamline diagnostic workflows (Burns et al., 2023).

Pneumonia remains a leading cause of mortality and morbidity among the elderly population worldwide, pneumonia accounts for approximately 90% of infection-related deaths in individuals aged 65 and over (GBD 2021 Lower Respiratory Infections and Antimicrobial Resistance Collaborators, 2024). The 28-day mortality rate of elderly inpatients with pneumonia often exceeds 18%, and if combined with underlying diseases, the mortality rate can rise to more than 30% (Erratum: Marked reduction in 28-day mortality among elderly patients with severe community-acquired pneumonia: post hoc analysis of a large randomized controlled trial [Corrigendum, 2020). However, the empirical medication adopted in its treatment often results in suboptimal therapeutic outcomes. Moreover, due to overlapping lesions, it is difficult to make a diagnosis solely through computed tomography (CT) (van Vugt et al., 2013; Cilloniz et al., 2016; Albano et al., 2020).Therefore, researchers have developed Pneumonia-Plus, a deep learning AI model for assisting the accurate classification of pneumonia based on CT images, using the HarDNet algorithm. Specifically, the model adopts an L-layer structure, adjusts with a channel weight (m = 1.6 in this study), and integrates four groups of HarD blocks. It can rapidly and accurately identify bacterial, fungal, and viral pneumonia, and the diagnostic performance of this model is significantly superior to that of the radiologists involved in the experiment, with sensitivities of 0.694 and 0.580 (Wang et al., 2023). Developed by the team of Jun Shao, the Multimodal Medical Information (MMI) system uses an attention architecture to fuse clinical text and imaging data. The system predicts pulmonary etiologies with high precision, drastically reducing diagnostic time for patients (Shao et al., 2024).

The elderly population represents a high-risk group for superficial fungal infections. Due to age-related immune decline and the presence of multiple comorbidities, these patients often experience more severe symptoms, prolonged treatment courses, and a higher prevalence of antifungal resistance (Maskan Bermudez et al., 2023). AI technology has brought revolutionary breakthroughs to fungal diagnosis, which is particularly aligned with the clinical needs of superficial fungal infections in the elderly. DL-based CNNs can perform intelligent analysis on various fungus-related images, they can identify the lesion characteristics of onychomycosis from macroscopic nail photos, and accurately locate fungal hyphae or spores in potassium hydroxide (KOH) stains and Periodic Acid Schiff (PAS) stained pathological sections. The diagnostic accuracy of some models for onychomycosis, a condition with a high incidence among the elderly, is even superior to that of dermatologists (Hasan Pour, 2025). Similar CNN architectures have been applied to parasite screening, significantly enhancing diagnostic efficiency by minimizing the time required for exhaustive manual slide examinations (Fuhad et al., 2020). What is more innovative is the technical approach combining single-cell Raman spectroscopy with AI algorithms. By extracting the biochemical fingerprint spectra of fungal cells, this approach can not only distinguish fungi and bacteria with 100% accuracy, but also achieve precise identification at the fungal species level. It does not rely on fungal culture, compressing the detection time from days to months required by traditional methods to within 1 hour (Xu et al., 2023), it greatly meets the clinical needs of elderly patients for rapid diagnosis and timely intervention.

### 4.3 AI in the treatment of infectious diseases

Tuberculosis (TB) is a disease susceptible to the elderly. The age-standardized incidence rate (ASIR) of TB among individuals aged over 60 years is approximately 190.44 per 100,000 population, and the ASIR of multidrug-resistant TB (MDR-TB) and extensively drug-resistant TB (XDR-TB) is also increasing year by year (Liu et al., 2025). Turon et al. developed an infectious disease analysis model targeting malaria and tuberculosis, which enables dose optimization of administered medications. This model uses machine learning for pharmacogene prioritisation. It selects 10 common infectious diseases and incorporates AI-predicted prioritised genes into the PBPK model. The results were then integrated into the NLME model, which showed that over 91% of the patient population achieved the target AUC (Turon et al., 2025). This holds significant importance for the elderly, as it can help avoid adverse reactions caused by traditional standard drug dosages.

Among women aged 65 years and older, over 10% have a history of urinary tract infections (UTIs), while this proportion surges to nearly 30% in individuals aged 85 years and above (Foxman et al., 2000; Öztürk and Murt, 2020; Chao et al., 2021). Shapiro Ben David et al. has developed an AI for diagnosis and treatment of UTIs. This tool comprises three core modules: a ML-based core algorithm for antibiotic resistance, a preset antibiotic prioritization system, and a patient characteristic analysis module, all of which have been integrated into the EHR system (Shapiro Ben David et al., 2025). This tool can assist clinicians in developing personalized antibiotic treatment plans for the elderly, thereby reducing the use of broad-spectrum antibiotics and slowing the development of antimicrobial resistance.

Although AI has significantly shortened diagnostic time and enabled precise drug administration, however, antimicrobial resistance challenges remain in the elderly (Othman et al., 2025). Intrinsic genetic mutations and transferrable antibiotic resistance genes (ARGs) lie at the core of antibiotic resistance development. Traditional alignment-based methods for ARG detection have inherent limitations, whereas AI enables the direct identification and classification of ARGs from genomic DNA sequences and plasmid sequences (Olatunji et al., 2024), this enables the rapid identification of genes contributing to drug resistance in older adults, thereby facilitating further clinical treatment. In addition, AI can also conduct an in-depth analysis of a dataset of antibiotic prescription records and, in combination with the Shapley explanation model, predict the potential emergence of antimicrobial resistance (Cavallaro et al., 2023). Notably, ML models are capable of predicting tuberculosis drug resistance with an accuracy exceeding 96%, thus supporting evidence-based clinical treatment decision-making (Pruthi et al., 2024). For the elderly, this can significantly reduce the risk of mismatched treatment and provide a strong guarantee for the rational clinical use of drugs in the early stage for the elderly.

Additionally, Wan et al. leveraged large-scale biomedical databases and employed ML and DL algorithm models to deeply explore the potential therapeutic value of existing drugs by constructing drug-target-disease association networks. This approach enabled the accurate prediction of new indications for approved drugs in common infectious diseases among the elderly (Wan et al., 2025). AI has also promoted the design of novel antibiotic (Popa et al., 2022; Catacutan et al., 2025; de la Fuente-Nunez and Collins, 2025) novel vaccine (Keshavarzi Arshadi et al., 2020), antimicrobial peptides (AMPs) (Ma et al., 2022; Talat and Khan, 2023; Wang et al., 2025) and antibacterial biomaterials (Jiang et al., 2025) against drug-resistant pathogens. Due to their strong antimicrobial activity, high targeting ability, and excellent safety profile, they can better address the problem of multidrug resistance (MDR) in elderly infectious diseases. AI can also predict the interactions between bacteriophages, bacteria, and hosts, thereby facilitating the design of novel phage therapy regimens more suitable for the elderly (Zhang et al., 2024), all of which provide new strategies for addressing drug-resistant bacterial infections in the elderly.

## 5 Outlook

When developing AI diagnostic and predictive tools for the elderly, the following key areas should be focused on in the future to enhance the applicability and reliability of the models.

### 5.1 Constructing geriatric-specific systematic training dataset

Future research should construct a systematic training dataset that incorporates features such as multimorbidity, multi-organ functional decline, immunosenescence, and chronic inflammation in the elderly population, to enhance the model’s generalization ability in various elderly care scenarios. At the same time, it is necessary to integrate clinical and non-clinical multi-dimensional predictive factors, fully considering the unique factors of the elderly, such as their daily living ability, cognitive function, care resources, and socioeconomic background.

### 5.2 Establishing an ethical and human-centered governance framework

In addition to the above technical requirements, in the advancement of AI-assisted diagnosis and treatment tools for the elderly, a concurrent effort must be made to establish a people-centered, secure, and trustworthy framework for data and ethical practices. Specifically, the following areas require enhanced development. First, an informed consent mechanism tailored to the characteristics of the elderly population should be established. For elderly individuals with significant variations in cognitive capacity, especially those with cognitive impairments or deficits, more inclusive and comprehensible consent procedures need to be developed. This ensures that their genuine intentions are fully respected and protected throughout the data collection and application process.Second, data privacy protection and anonymization technologies must be strengthened. During complex modeling processes, verifiable de-identification and encryption techniques should be employed to prevent the re-identification of personal identities and sensitive information, effectively mitigating the risk of privacy breaches. Third, a people-centered approach should be upheld to leverage the auxiliary and collaborative value of AI. The design and deployment of tools must prioritize supporting physician-patient communication and respecting the elderly’s life preferences, avoiding the replacement of humanistic care with automated decision-making. While enhancing technical efficiency, the patient’s quality of life and personal autonomy should be placed at the core, achieving an organic balance between technological empowerment and humanized care.

### 5.3 AI in the diagnosis of parasitic infections

The application of AI to parasitic infection diagnosis warrants dedicated attention, as this domain presents a distinctive set of challenges and opportunities that differ substantially from bacterial or viral infections, particularly in the elderly, who bear a disproportionate burden of parasitic diseases such as toxoplasmosis, cryptosporidiosis, and reactivated latent parasitic infections due to immunosenescence (Yogesh and Garg, 2024). Taking malaria as an example, current AI based predictive models still face several key limitations. Most models struggle to accurately predict the severity of outbreaks and can only determine the timing and location of their occurrence. Malaria transmission is influenced by multiple interacting factors including climate, human migration, and healthcare seeking behavior. Existing models often incorporate only a subset of these variables, resulting in incomplete predictions. Furthermore, the lack of high resolution data, unreliable data quality, and low adoption rates of these models in grassroots disease control practices further constrain their practical utility. The shortage of local modeling expertise in Africa also limits the continued optimization and localized deployment of these tools (Nordling, 2023).

Future directions in the application of AI to parasitic disease diagnosis include the establishment of multi species parasite image repositories that are geographically diverse, cross regional, and uniformly annotated. There is also a growing need to deploy lightweight models optimized for edge computing on smartphone equipped microscopes or portable devices, thereby facilitating point of care screening in resource limited settings. Furthermore, leveraging pretrained models via transfer learning or few shot learning strategies can effectively address data scarcity for neglected or rare parasitic infections and support rapid adaptation to newly endemic regions (Zhang et al., 2022; Tunali, 2026). Through continuous refinement in these areas, AI can truly serve as a reliable enabler for improving the health and well-being of the elderly, while simultaneously safeguarding their rights and privacy.

## 6 Conclusion

Traditional prevention, diagnosis, treatment, and control paradigms face inherent limitations in addressing the complex, multifactorial clinical challenges characteristic of older adults, including age-related organ dysfunction, immunosenescence and inflammaging, chronic comorbidities, psychosocial factors. In contrast, AI is increasingly being integrated across the continuum of infectious disease management in this population. Leveraging advanced methodologies, including ML, such as ensemble algorithms and XGBoost, CNNs for imaging or time series analysis, AI systems demonstrate robust potential to improve diagnostic precision, therapeutic personalization. Despite the ongoing challenges such as algorithmic bias, insufficient representation of the elderly in training data, and limited real-world validation, AI has demonstrated significant potential in the full process of diagnosis and treatment of infectious diseases in the elderly. To fully realize this potential, it is necessary to carry out structured AI literacy training at the clinical level to enhance the understanding of healthcare workers regarding model interpretability and scenario-based applications. At the same time, institutions should invest in building interoperable technical infrastructure and establish an ethics-based governance framework to systematically promote the reliable integration and effective implementation of AI in the diagnosis and treatment of infectious diseases in the elderly.
